# Association between Abortion and All-Cause and Cause-Specific Premature Mortality: A Prospective Cohort Study from the UK Biobank

**DOI:** 10.34133/hds.0147

**Published:** 2024-07-15

**Authors:** Shaohua Yin, Yingying Yang, Qin Wang, Wei Guo, Qian He, Lei Yuan, Keyi Si

**Affiliations:** ^1^ Department of Medical Engineering, Peking University Third Hospital, Beijing, China.; ^2^ Clinical Research Center, Shanghai Key Laboratory of Maternal Fetal Medicine, Shanghai Institute of Maternal-Fetal Medicine and Gynecologic Oncology, Shanghai First Maternity and Infant Hospital, School of Medicine, Tongji University, Shanghai, China.; ^3^ Department of Health Management, Naval Medical University, Shanghai, China.; ^4^ Department of Military Health Statistics, Naval Medical University, Shanghai, China.; ^5^ Department of Environmental Health, School of Public Health, Shanghai Jiao Tong University School of Medicine, Shanghai, China.

## Abstract

**Background:** Concerns have been raised about the increasing prevalence of both spontaneous and induced abortions worldwide, yet their effect on premature mortality remains poorly understood. We aimed to examine the associations between abortion and all-cause and cause-specific premature mortality, and the potential effect modification by maternal characteristics. **Methods:** Women aged 39 to 71 years at baseline (2006 to 2010) with prior pregnancies were derived from the UK Biobank and categorized as no abortion history, spontaneous abortion alone, induced abortion alone, and both spontaneous and induced abortions. All-cause and cause-specific mortality were ascertained through linkage to death certificate data, with premature death defined as occurring before the age of 70. **Results:** Of the 225,049 ever gravid women, 43,418 (19.3%) reported spontaneous abortion alone, 27,135 (12.1%) reported induced abortion alone, and 10,448 (4.6%) reported both spontaneous and induced abortions. During a median of 14.4 years of follow-up, 5,353 deaths were recorded, including 3,314 cancer-related and 1,444 cardiovascular deaths. Compared with no abortion history, spontaneous abortion alone was associated with an increased risk of all-cause premature mortality (adjusted hazard ratio [aHR] 1.10, 95% confidence interval [CI] 1.02 to 1.17), and induced abortion alone was associated with increased risks of all-cause (aHR 1.12, 95% CI 1.04 to 1.22) and cardiovascular mortality (aHR 1.27, 95% CI 1.09 to 1.48). The aHRs for all-cause and cardiovascular mortality were higher for recurrent abortions, whether spontaneous or induced (*P*_trend_ < 0.05). The increased risk of all-cause mortality associated with induced abortion was higher in women with hypertensive disorders of pregnancy than in those without (40% vs. 9%, *P*_interaction_ = 0.045). **Conclusions:** Either spontaneous or induced abortion alone was associated with an increased risk of premature mortality, with induced abortion alone particularly linked to cardiovascular death. Future studies are encouraged to explore the underlying mechanisms.

## Introduction

Globally, non-communicable diseases (NCDs) are the predominant cause of premature death before 70 years of age, accounting for 60.1% of all premature deaths in 2019 and presenting an ongoing challenge to healthy aging [1,2]. According to estimates from the World Health Organization (WHO), the number of premature deaths due to NCDs in women has risen from 5.8 million in 2000 to 6.8 million in 2019 worldwide [1,2], and such deaths from the UK was 46,654 in 2019, exceeding several countries including France, Italy, and Canada [1]. To achieve the Sustainable Development Goal of reducing premature mortality from NCDs by one-third before 2030, it is crucial to raise public awareness regarding the risk factors associated with premature mortality [3]. While well-established factors such as smoking, overweight/obesity, unhealthy diet, and physical inactivity continue to be predominant risk factors for premature mortality, emerging evidence suggests that certain reproductive traits, including infertility, parity, and pregnancy complications, may also play a role in long-term morbidity related to premature mortality [4–6].

Abortion, encompassing both spontaneous (miscarriage or natural pregnancy loss) and induced (voluntary termination of pregnancy by medical or surgical means) pregnancy losses before viability, is a prevalent global issue, resulting in an estimated 96 million cases annually worldwide [7–10]. The number of abortions per 1,000 women of reproductive age varied widely, ranging from 10 in the United Arab Emirates to 48 in Afghanistan for spontaneous abortion [11], and from 5 in Singapore to 80 in Georgia for induced abortion [12], attributing to diverse healthcare systems, socio-economic factors, cultural norms, and access to maternal care [12]. About 15 and 26 in every 100 pregnancies in the UK ended in spontaneous and induced abortion, respectively [8,10–13]. Abortion is often physiologically linked to other diseases and disorders, such as embryonic chromosomal errors and endometrial defects [14,15], and has been associated with increased risks of prelabor rupture of membranes, placental dysfunction disorders, psychological disorders, and perinatal mortality [16–18]. Two large-scale cohort studies conducted in the United States and Finland suggested a higher risk of maternal mortality associated with spontaneous or induced abortion; however, evidence linking abortion with maternal premature mortality remains extremely scarce [19,20].

In this study, we aimed to investigate the association of abortion (spontaneous abortion alone, induced abortion alone, and both spontaneous and induced abortion) with all-cause and cause-specific premature mortality in women with reproductive history. We also explored the potential effect modifications of sociodemographic factors, lifestyle behaviors, and medical conditions in the association.

## Methods

### Study design and participants

**Fig. 1.**
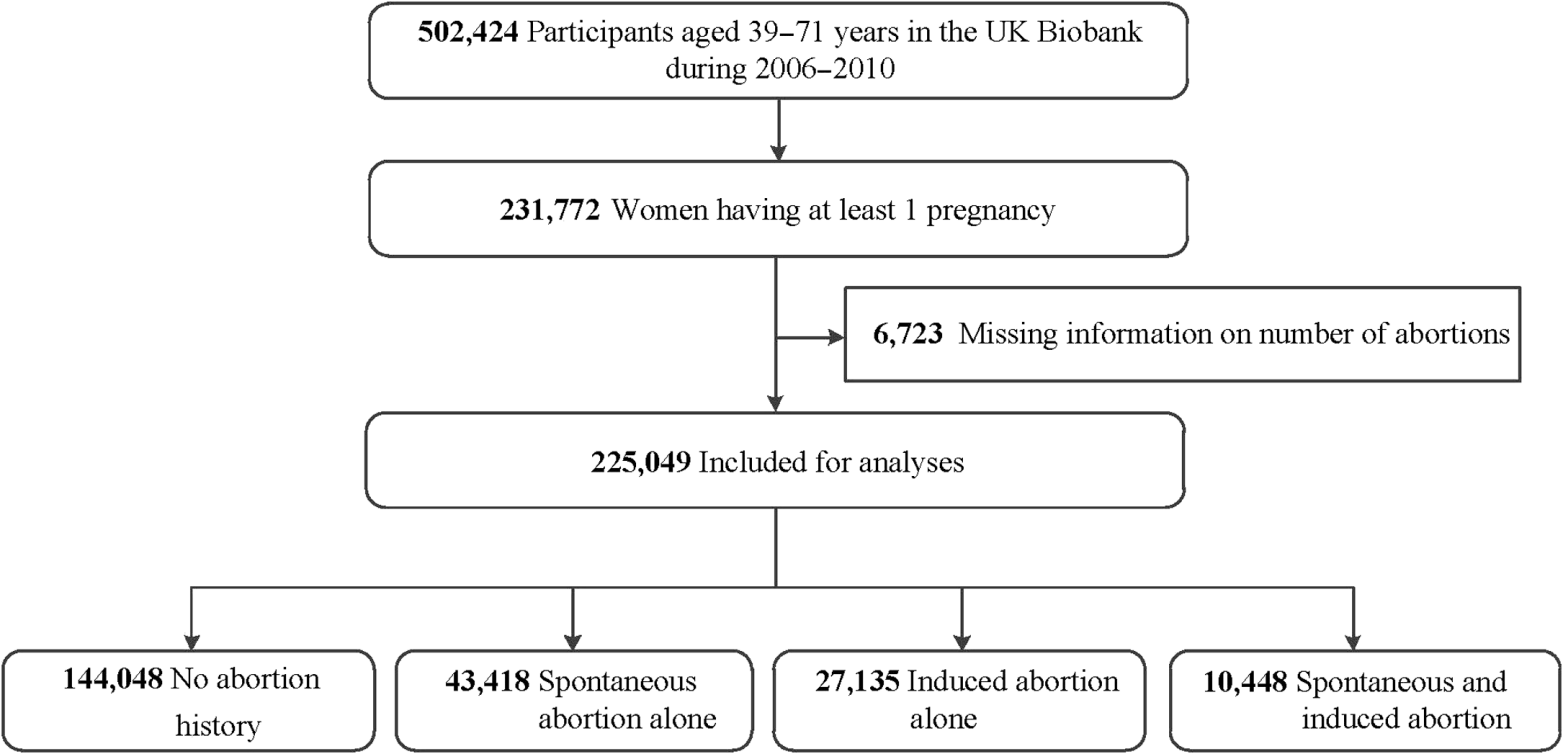
Participant enrollment flowchart.

This nationwide, population-based, prospective cohort study used data from the UK Biobank (UKB), which was designed to provide insights into disease and health status of volunteer-based residents in the UK [21]. Initially, over 0.5 million men and women aged between 39 and 71 years were recruited from 22 dedicated assessment centers across England, Scotland, and Wales between 2006 and 2010. Participants were asked to complete a touch-screen questionnaire, have physical and functional measurements, and provide biological samples at enrollment. Details of the study design can be found at www.ukbiobank.ac.uk. Of the 273,297 female participants, 231,772 (84.8%) reported having a history of at least one pregnancy. We excluded 6,723 women with missing information regarding the number of spontaneous or induced abortions, resulting in 225,049 women included in our analyses (Fig. 1).

### Exposure and outcome

Exposures of interest were type and number of abortion, which were assessed using several questions on the questionnaire—“Have you ever had any stillbirths, spontaneous miscarriages or terminations?”, “How many spontaneous miscarriages?” and “How many terminations?”, in addition to date of O03 (spontaneous abortion), O04 (medical abortion), O05 (other abortion), and O06 (unspecified abortion) on the medical records that mapped the 3-character codes in the International Classification of Diseases, 10th version (ICD-10). Abortion was classified into 4 categories: no abortion history, spontaneous abortion alone (defined as a miscarriage or natural pregnancy loss), induced abortion alone (defined as a voluntary pregnancy termination, including medical and surgical abortion), and both spontaneous and induced abortions.

Outcome of this study was all-cause death, including mortality due to cardiovascular disease, cancer, and others (such as respiratory disease, neurological disease, or accidents). Dates of death were extracted from death certificates held by the National Health Service (NHS) Information Centre (England and Wales) and the NHS Central Register (Scotland). Cardiovascular disease was defined using I00 to I99 and cancer using C00 to C97 in the ICD-10 [22]. Age at the time of death was calculated as the difference between the date of death and date of birth, with premature death defined as women who died before the age of 70 [19]. Women who died at or beyond the age of 70 or who were event-free at the end of the study were considered censored cases in the analysis [23].

### Covariate ascertainment

Information on sociodemographic characteristics, lifestyle behaviors, medical history, use of medication, and family history was derived from nurse-led self-report questionnaires. In our analyses, age (difference between date of enrollment and date of birth) was categorized into 4 groups (≤54, 55 to 59, 60 to 64, and ≥65 years). Ethnicity originally reported as White, mixed, Asian or Asian British, Black or Black British, Chinese, or other ethnic group was recategorized as White and non-White. Education was categorized as having college/university degree or other qualifications. Annual household income was divided into <£18,000, £18,000 to 51,999, £52,000 to 100,000, and >£100,000. Smoking status was reported as yes or no, and drinking status was reported as never, former, or current. Physical activity was assessed by the International Physical Activity Questionnaire, with average weekly energy expenditure calculated and categorized into tertiles. Diet score was constructed based on the consumption of fruits, vegetables, fish, processed meat, unprocessed red meat, whole grains, and refined grains obtained from standardized food frequency questionnaires and then categorized into tertiles [24]. Pre-existing doctor-diagnosed diabetes or cancer, previous use of oral contraceptives or hormone-replacement therapy, parental history of myocardial infarction or stroke, and menopause status were self-reported as yes or no. Reproductive conditions including gestational diabetes (O24), hypertensive disorders of pregnancy (HDP, O10 to O16), hemorrhage during early pregnancy (O20), endometriosis (N80), and ectopic pregnancy (O00) were defined by the ICD-10 codes using the “first-occurrence” data fields. Body mass index (BMI) was calculated as weight in kilograms divided by the square of height in meters, which were measured by trained nurses at enrollment, and classified into underweight (<18.5 kg/m^2^), normal weight (18.5 to 24.9 kg/m^2^), overweight (25.0 to 29.9 kg/m^2^), and obesity (≥30.0 kg/m^2^) using the WHO criterion.

### Statistical analysis

Baseline characteristics are presented as proportions for categorical variables and means (standard deviations [SDs]) for continuous variables. Differences in baseline characteristics between types of abortion were examined by Chi-square test for categorical variables.

Cox proportional hazards regression models [25] were used to estimate the hazard ratios (HRs) and 95% confidence intervals (CIs) for premature mortality across 4 categories of abortion. Proportional hazards assumption was graphically tested using a log cumulative hazard plot, where the logarithm of time was plotted against the estimated log cumulative hazard for the compared groups. The observed curves were found to be approximately parallel, validating the assumption of proportional hazards. Follow-up for all participants commenced from the date of enrollment until the date of death ascertainment or the censor date (2022 December 1). The association between abortion and all-cause premature mortality was firstly assessed, followed by analyses on subtypes of premature death from cardiovascular disease, cancer, and other causes. Multivariable models were adjusted for age, BMI, ethnicity, education, average household income, smoking status, drinking status, physical activity, diet score, pre-existing diabetes or cancer, use of oral contraceptives or hormone treatment, menopausal status, gestational diabetes, HDP, hemorrhage during early pregnancy, endometriosis, ectopic pregnancy, and parental history of myocardial infarction or stroke. Furthermore, we calculated the attributable risk proportion to estimate the premature death burden due to various abortions [26]. Interactions between BMI, smoking status, endometriosis, and HDP and abortion on the risk of premature death were examined by adding an interaction term to the multivariable model. Numerical variables with missing data were transformed into categorical variables, where the categorical values were encoded into numbers ranging from 0 to a positive integer, and missing data were coded as “9” and classified as a separate category.

To test the robustness of the findings, several sensitivity analyses were conducted. First, we used the multiple imputation by chained equations approach (SAS PROC MI, followed by PROC MIANALYZE) to impute missing values of potential confounding variables [27], with 5 imputed datasets. The multivariable models were then reevaluated with the pooled results. Second, we restricted the analysis to women who were cancer-free at baseline (*n* = 203,770). Additional sensitivity analysis was performed in women who had given a live birth (*n* = 179,692), or in those who were aged greater than 50 years at baseline (*n* = 176,028).

A 2-sided *P* value of 0.05 or less indicated the significance. All statistical analyses were conducted using SAS version 9.4 (SAS Institute Inc.).

### Role of the funding source

The funders had no role in study design, data collection, data analysis, data interpretation, or writing of the report.

## Results

Of the 225,049 ever gravid women included, 43,418 (19.3%) experienced spontaneous abortion alone, 27,135 (12.1%) experienced induced abortion alone, and 10,448 (4.6%) experienced both spontaneous and induced abortions (Fig. 1). Table 1 presents the characteristics of women across type of abortion. Women with no history of abortion were more likely to have a higher proportion of older age, and a higher rate of the use of hormone treatment and HDP. Current smoking and drinking, as well as having gestational diabetes, endometriosis, use of oral contraceptives, baseline cancer, and ectopic pregnancy were more common in women who experienced abortions compared to those with no history of abortion (Table 1). Proportions of adherence to a healthy diet and adequate physical activity were higher among women with a history of abortion than those without (Table 1).

**Table 1.** General characteristics according to type of abortion either at baseline or during follow-up among 225,049 women. Percentages may not sum to 100 due to rounding.

Characteristics	No abortion history (*n* = 144,048)	Spontaneous abortion alone (*n* = 43,418)	Induced abortion alone (*n* = 27,135)	Spontaneous and induced abortion (*n* = 10,448)	*P* values
**Age at baseline (years)**					<0.001
≤54	47,644 (33.08)	16,555 (38.13)	13,800 (50.86)	5,506 (52.70)	
55–59	27,297 (18.95)	7,900 (18.20)	5,168 (19.05)	1,817 (17.39)	
60–64	39,024 (27.09)	10,699 (24.64)	5,211 (19.20)	1,968 (18.84)	
≥65	30,083 (20.88)	8,264 (19.03)	2,956 (10.89)	1,157 (11.07)	
**BMI at baseline (kg/m^2^)**					<0.001
<18.5	906 (0.63)	298 (0.69)	194 (0.71)	61 (0.58)	
18.5–24.9	54,392 (37.76)	16,108 (37.10)	10,849 (39.98)	4,147 (39.69)	
25.0–29.9	54,427 (37.78)	16,158 (37.21)	9,642 (35.53)	3,641 (34.85)	
≥30.0	33,731 (23.42)	10,649 (24.53)	6,293 (23.19)	2,551 (24.42)	
Missing	592 (0.41)	205 (0.47)	157 (0.58)	48 (0.46)	
**Ethnicity**					<0.001
White	137,372 (95.37)	41,289 (95.10)	24,589 (90.62)	9,282 (88.84)	
Non-White	6,350 (4.41)	2,001 (4.61)	2,465 (9.08)	1,137 (10.88)	
Missing	326 (0.23)	128 (0.29)	81 (0.30)	29 (0.28)	
**Education**					<0.001
College or university degree	12,782 (8.87)	4,289 (9.88)	3,593 (13.24)	1,434 (13.73)	
Others	131,266 (69.02)	39,129 (71.82)	23,542 (73.08)	9,014 (72.50)	
Missing	31,844 (22.11)	7,946 (18.30)	3,711 (13.68)	1,439 (13.77)	
**Smoking status**					<0.001
No	67,105 (46.59)	19,239 (44.31)	9,102 (33.54)	3,562 (34.09)	
Yes	76,359 (53.01)	24,043 (55.38)	17,966 (66.21)	6,856 (65.62)	
Missing	584 (0.41)	136 (0.31)	67 (0.25)	30 (0.29)	
**Drinking status**					<0.001
Never	8,793 (6.10)	2,626 (6.05)	1,068 (3.94)	506 (4.84)	
Previous	5,116 (3.55)	1,669 (3.84)	1,086 (4.00)	418 (4.00)	
Current	129,990 (90.24)	39,087 (90.02)	24,959 (91.98)	9,513 (91.05)	
Missing	149 (0.10)	36 (0.08)	22 (0.08)	11 (0.11)	
**Physical activity, MET minutes per week**					<0.001
Tertile 1	35,896 (24.92)	11,153 (25.69)	7,305 (26.92)	2,738 (26.21)	
Tertile 2	36,171 (25.11)	11,111 (25.59)	7,225 (26.63)	2,747 (26.29)	
Tertile 3	37,263 (25.87)	11,175 (25.74)	7,526 (27.74)	2,955 (28.28)	
Missing	34,718 (24.10)	9,979 (22.98)	5,079 (18.72)	2,008 (19.22)	
**Diet score**					<0.001
Tertile 1	26,445 (18.36)	8,096 (18.65)	4,832 (17.81)	1,859 (17.79)	
Tertile 2	59,616 (41.39)	17,825 (41.05)	10,737 (39.57)	4,111 (39.35)	
Tertile 3	49,769 (34.55)	14,861 (34.23)	9,741 (35.90)	3,742 (35.82)	
Missing	8,218 (5.71)	2,636 (6.07)	1,825 (6.73)	736 (7.04)	
**Diabetes at baseline**					<0.001
No	138,245 (95.97)	41,397 (95.35)	26,185 (96.50)	10,015 (95.86)	
Yes	5,486 (3.81)	1,922 (4.43)	897 (3.31)	412 (3.94)	
Missing	317 (0.22)	99 (0.23)	53 (0.20)	21 (0.20)	
**Cancer at baseline**					0.026
No	130,409 (90.53)	39,192 (90.27)	24,673 (90.93)	9,496 (90.89)	
Yes	13,106 (9.10)	4,036 (9.30)	2,359 (8.69)	906 (8.67)	
Missing	533 (0.37)	190 (0.44)	103 (0.38)	46 (0.44)	
**Use of oral contraceptives**					<0.001
Never	27,026 (18.76)	7,700 (17.73)	2,812 (10.36)	1,228 (11.75)	
Ever	116,733 (81.04)	35,637 (82.08)	24,299 (89.55)	9,200 (88.06)	
Missing	289 (0.20)	81 (0.19)	24 (0.09)	20 (0.19)	
**Use of hormone treatment**					<0.001
Never	84,908 (58.94)	25,826 (59.48)	17,702 (65.24)	6,903 (66.07)	
Ever	58,691 (40.74)	17,453 (40.20)	9,371 (34.53)	3,510 (33.59)	
Missing	449 (0.31)	139 (0.32)	62 (0.23)	35 (0.33)	
**Gestational diabetes**					<0.001
No	143,088 (99.33)	42,996 (99.03)	26,922 (99.22)	10,316 (98.74)	
Yes	800 (0.56)	362 (0.83)	143 (0.53)	116 (1.11)	
Missing	160 (0.11)	60 (0.14)	70 (0.26)	16 (0.15)	
**Hypertensive disorders of pregnancy**					<0.001
No	111,502 (77.41)	33,433 (77.00)	21,865 (80.58)	8,372 (80.13)	
Yes	32,546 (22.59)	9,985 (23.00)	5,270 (19.42)	2,076 (19.87)	
**Hemorrhage during early pregnancy**					<0.001
No	143,668 (99.74)	42,728 (98.41)	27,006 (99.52)	10,207 (97.69)	
Yes	380 (0.26)	690 (1.59)	129 (0.48)	241 (2.31)	
**Endometriosis**					<0.001
No	139,476 (96.83)	41,665 (95.96)	26,198 (96.55)	10,005 (95.76)	
Yes	4,572 (3.17)	1,753 (4.04)	937 (3.45)	443 (4.24)	
**Ectopic pregnancy**					<0.001
No	143,715 (99.77)	43,062 (99.18)	26,987 (99.45)	10,315 (98.73)	
Yes	333 (0.23)	356 (0.82)	148 (0.55)	133 (1.27)	
**Parental history of myocardial infarction or stroke**					<0.001
No	96,988 (67.33)	28,794 (66.32)	17,917 (66.03)	6,739 (64.50)	
Yes	21,671 (15.04)	7,029 (16.19)	4,000 (14.74)	1,592 (15.24)	
Missing	25,389 (17.63)	7,595 (17.49)	5,218 (19.23)	2,117 (20.26)	

BMI, body mass index; MET, metabolic equivalent of task.

**Fig. 2.**
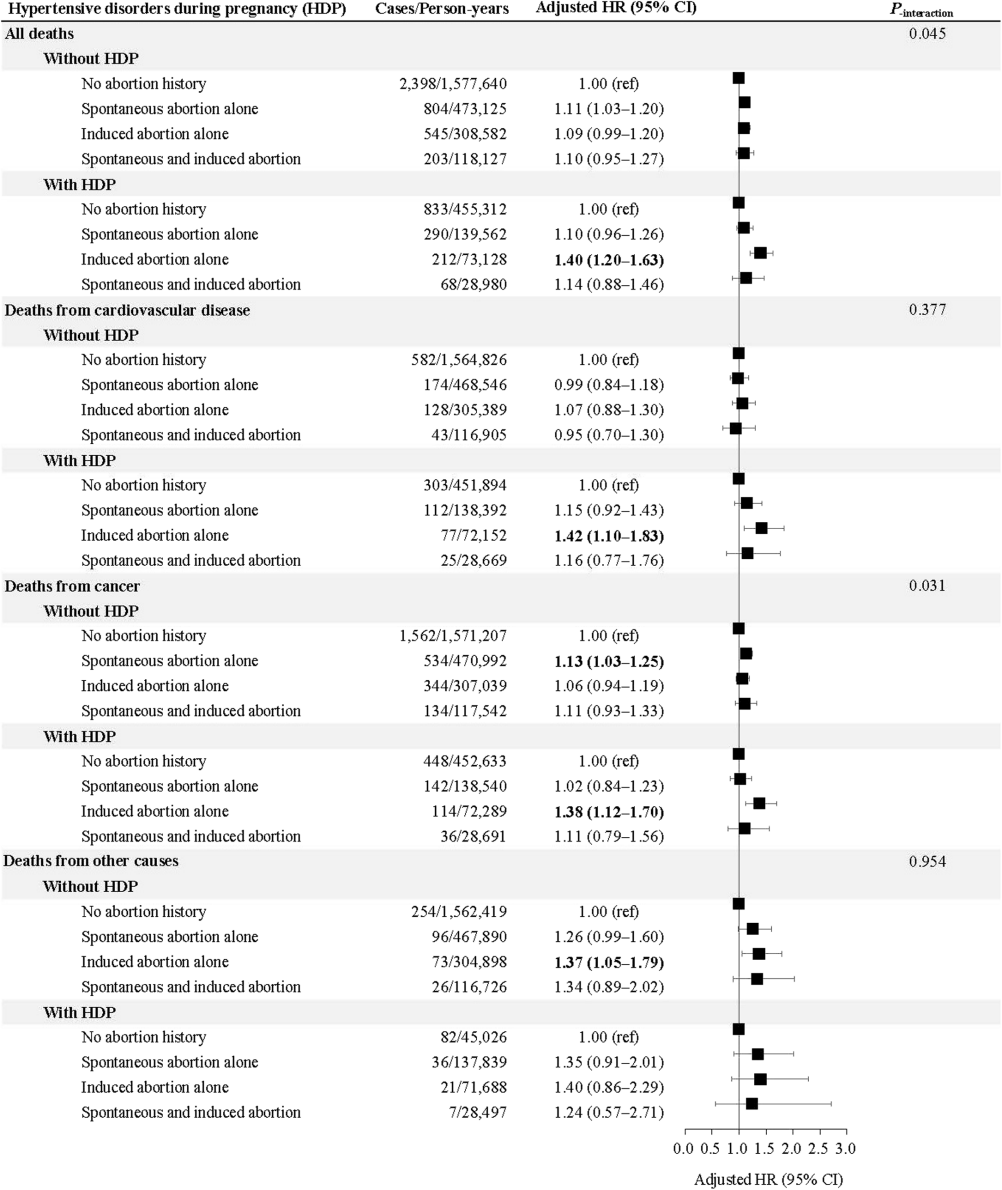
Adjusted hazard ratios (aHRs) and 95% confidence intervals (CIs) for premature mortality (before age 70 years) according to type of abortion, stratified by hypertensive disorder of pregnancy (HDP). Multivariable models were adjusted for age, BMI, ethnicity, education, average household income, smoking status, drinking status, physical activity, diet score, diabetes at baseline, cancer at baseline, use of oral contraceptives, use of hormone treatment, menopausal status, gestational diabetes, hemorrhage during early pregnancy, endometriosis, ectopic pregnancy, and parental history of myocardial infarction or stroke.

During a median follow-up of 14.4 (interquartile range 1.4) years, 5,353 premature deaths were recorded, including 3,314 (61.9%) deaths from cancer, 1,444 (27.0%) from cardiovascular disease, and 595 (11.1%) from all other causes, resulting in a premature mortality rate of 1.69 per 1,000 person-years. The corresponding rate for women with no history of abortion, spontaneous abortion alone, induced abortion alone, and both spontaneous and induced abortions were 1.59, 1.79, 1.98, and 1.84 per 1,000 person-years, respectively. After adjustment for confounders, women who experienced spontaneous abortion alone (adjusted hazard ratio [aHR] 1.10, 95% CI 1.02 to 1.17) and induced abortion alone (aHR 1.12, 95% CI 1.04 to 1.22) had an increased risk of all-cause premature mortality compared to women with no abortion history. The attributable risk proportions for spontaneous abortion alone and induced abortion alone were 8.68% and 10.87%, respectively (Table 2). Furthermore, a monotonic increase in aHRs for all-cause premature mortality was observed based on the number of spontaneous abortions and induced abortions (*P*_trend_ <0.001). For example, women with one induced abortion had an aHR of 1.40 (95% CI 1.28 to 1.53), women with 2 induced abortions had an aHR of 1.56 (95% CI 1.30 to 1.88), and women with 3 or more induced abortions had an aHR of 1.50 (95% CI 1.02 to 2.20) (Table 3). Similar patterns were observed for women aged greater than 50 years at baseline (Table S1). Additionally, the increased risk of all-cause mortality associated with induced abortion was higher in women with HDP than without (40% vs. 9%, *P*_interaction_ = 0.045) (Fig. 2). However, there was no effect modification of the associations between induced abortion alone and premature mortality by BMI at baseline, smoking status, or endometriosis (Table S2). No associations were found for women with both spontaneous and induced abortions.

**Table 2.** Hazard ratios (HRs) and 95% confidence intervals (CIs) for all-cause and cause-specific premature mortality (before age 70 years) according to type of abortion. Bold indicates that *P* values less than 0.05 were considered statistically significant.

	Cases/Person-years	Crude model		Multivariable model^a^	
		HR (95% CI)	AR%	HR (95% CI)	AR%
All deaths					
No abortion history	3,231/2,032,953	1.00 (ref)		1.00 (ref)	
Spontaneous abortion alone	1,094/612,687	**1.12 (1.05–1.20)**	11.03	**1.10 (1.02–1.17)**	8.68
Induced abortion alone	757/381,710	**1.25 (1.15–1.35)**	19.81	**1.12 (1.04–1.22)**	10.87
Spontaneous and induced abortion	271/147,107	**1.16 (1.02–1.31)**	13.64	1.06 (0.94–1.20)	5.84
Deaths from cardiovascular disease					
No abortion history	885/2,016,721	1.00 (ref)		1.00 (ref)	
Spontaneous abortion alone	286/606,938	1.07 (0.94–1.23)	6.72	1.05 (0.92–1.20)	4.94
Induced abortion alone	205/377,541	**1.24 (1.06–1.44)**	19.03	**1.27 (1.09–1.48)**	21.20
Spontaneous and induced abortion	68/145,574	1.06 (0.83–1.36)	5.93	1.07 (0.83–1.37)	6.54
Deaths from cancer					
No abortion history	2,010/2,023,840	1.00 (ref)		1.00 (ref)	
Spontaneous abortion alone	676/609,532	**1.12 (1.02–1.22)**	10.71	1.11 (0.98–1.21)	9.83
Induced abortion alone	458/379,328	**1.21 (1.09–1.34)**	17.36	**1.18 (1.07–1.31)**	15.33
Spontaneous and induced abortion	170/146,233	1.17 (1.00–1.36)	14.53	1.15 (0.98–1.34)	12.89
Deaths from other causes					
No abortion history	336/2,012,686	1.00 (ref)		1.00 (ref)	
Spontaneous abortion alone	132/605,728	**1.30 (1.07–1.59)**	23.31	**1.29 (1.06–1.58)**	22.72
Induced abortion alone	94/376,586	**1.49 (1.19–1.87)**	32.89	**1.47 (1.16–1.85)**	31.83
Spontaneous and induced abortion	33/145,223	1.36 (0.95–1.94)	26.31	1.37 (0.96–1.97)	27.17

AR%, attributable risk proportion; HR, hazard ratio; 95% CI, 95% confidence interval.

^a^ Multivariable models were adjusted for age, BMI, ethnicity, education, average household income, smoking status, drinking status, physical activity, diet score, diabetes at baseline, cancer at baseline, use of oral contraceptives, use of hormone treatment, menopausal status, gestational diabetes, hypertensive disorders of pregnancy, hemorrhage during early pregnancy, endometriosis, ectopic pregnancy, and parental history of myocardial infarction or stroke.

**Table 3.** Hazard ratios (HRs) and 95% confidence intervals (CIs) for all-cause and cause-specific premature mortality according to number of abortions. Bold indicates that *P* values less than 0.05 were considered statistically significant.

	Cases/Person-years	Crude model		Multivariable model^a^	
		HR (95% CI)	*P*_trend_	HR (95% CI)	*P*_trend_
**All deaths**					
Spontaneous abortion alone			<0.001		<0.001
None	3,231/2,032,953	1.00 (ref)		1.00 (ref)	
1	732/445,829	1.08 (1.00–1.17)		1.04 (0.96–1.13)	
2	227/108,948	**1.39 (1.22–1.59)**		**1.26 (1.10–1.44)**	
≥3	135/57,909	**1.60 (1.35–1.90)**		**1.36 (1.14–1.61)**	
Induced abortion alone			<0.001		<0.001
None	3,231/2,032,953	1.00 (ref)		1.00 (ref)	
1	614/310,363	**1.46 (1.34–1.59)**		**1.40 (1.28–1.53)**	
2	117/56,706	**1.68 (1.40–2.02)**		**1.56 (1.30–1.88)**	
≥3	26/14,642	**1.58 (1.07–2.32)**		**1.50 (1.02–2.20)**	
Spontaneous and induced abortion			<0.001		0.074
None	3,231/2,032,953	1.00 (ref)		1.00 (ref)	
2	137/82,280	**1.25 (1.05–1.48)**		1.02 (0.86–1.21)	
≥3	134/64,827	**1.66 (1.40–1.98)**		**1.20 (1.01–1.44)**	
**Deaths from cardiovascular disease**					
Spontaneous abortion alone			<0.001		0.011
None	885/2,016,721	1.00 (ref)		1.00 (ref)	
1	176/441,814	0.95 (0.81–1.12)		0.92 (0.78–1.08)	
2	61/107,839	**1.37 (1.06–1.78)**		1.20 (0.92–1.56)	
≥3	49/57,285	**2.13 (1.60–2.84)**		**1.70 (1.27–2.27)**	
Induced abortion alone			<0.001		<0.001
None	885/2,016,721	1.00 (ref)		1.00 (ref)	
1	171/307,004	**1.50 (1.27–1.76)**		**1.41 (1.20–1.67)**	
2	25/56,015	1.33 (0.90–1.99)		1.13 (0.76–1.69)	
≥3	9/14,521	**2.04 (1.06–3.94)**		**1.75 (1.01–3.39)**	
Spontaneous and induced abortion			0.009		0.695
None	885/2,016,721	1.00 (ref)		1.00 (ref)	
2	31/81,504	1.04 (0.73–1.48)		0.86 (0.60–1.23)	
≥3	37/64,070	**1.70 (1.22–2.36)**		1.20 (0.86–1.67)	
**Deaths from cancer**					
Spontaneous abortion alone			0.145		0.070
None	2,010/2,023,840	1.00 (ref)		1.00 (ref)	
1	464/443,840	1.10 (0.99–1.22)		1.06 (0.96–1.17)	
2	145/108,345	**1.43 (1.21–1.69)**		**1.31 (1.11–1.55)**	
≥3	67/57,347	**1.28 (1.00–1.63)**		1.12 (0.87–1.42)	
Induced abortion alone			<0.001		<0.001
None	2,010/2,023,840	1.00 (ref)		1.00 (ref)	
1	364/308,371	**1.38 (1.24–1.55)**		**1.31 (1.17–1.47)**	
2	79/56,398	**1.81 (1.45–2.27)**		**1.69 (1.35–2.12)**	
≥3	15/14,559	1.45 (0.87–2.41)		1.42 (0.85–2.36)	
Spontaneous and induced abortion			<0.001		0.171
None	2,010/2,023,840	1.00 (ref)		1.00 (ref)	
2	91/81,889	**1.33 (1.07–1.64)**		1.08 (0.87–1.33)	
≥3	79/64,343	**1.57 (1.26–1.97)**		1.15 (0.92–1.45)	
**Deaths from other causes**					
Spontaneous abortion alone			<0.001		0.008
None	336/2,012,686	1.00 (ref)		1.00 (ref)	
1	92/441,155	**1.30 (1.04–1.64)**		**1.27 (1.00–1.59)**	
2	21/107,533	1.24 (0.80–1.92)		1.10 (0.71–1.71)	
≥3	19/57,040	**2.16 (1.36–3.43)**		**1.82 (1.14–2.91)**	
Induced abortion alone			<0.001		0.053
None	336/2,012,686	1.00 (ref)		1.00 (ref)	
1	79/306,221	**1.79 (1.40–2.29)**		**1.42 (1.10–1.82)**	
2	13/55,885	**1.77 (1.02–3.09)**		1.23 (0.70–2.15)	
≥3	2/14,480	1.15 (0.29–4.60)		0.77 (0.19–3.13)	
Spontaneous and induced abortion			0.002		0.177
None	336/2,012,686	1.00 (ref)		1.00 (ref)	
2	15/81,355	1.30 (0.78–2.18)		1.06 (0.63–1.79)	
≥3	18/63,868	**2.13 (1.32–3.42)**		1.46 (0.90–2.37)	

AR%, attributable risk proportion; HR, hazard ratio; 95% CI, 95% confidence interval.

^a^ Multivariable models were adjusted for age, BMI, ethnicity, education, average household income, smoking status, drinking status, physical activity, diet score, diabetes at baseline, cancer at baseline, use of oral contraceptives, use of hormone treatment, menopausal status, gestational diabetes, hypertensive disorders of pregnancy, hemorrhage during early pregnancy, endometriosis, ectopic pregnancy, and parental history of myocardial infarction or stroke.

In the analyses of cause-specific premature death, women who experienced induced abortion alone had a significantly increased risk of death from cardiovascular disease, with an adjusted HR of 1.27 (95% CI 1.09 to 1.48), and a notable 21.20% attributable risk proportion (Table 2). The risk of premature death from cardiovascular disease increased with the number of induced abortions experienced. Specifically, women with one induced abortion had a 41% greater risk (95% CI 1.20 to 1.67), and women with 3 or more induced abortions had a 75% greater risk (95% CI 1.01 to 3.39), compared to women with no history of abortion (Table 3). Although there was no association between spontaneous abortion and risk of premature death from cardiovascular disease, women with 3 or more spontaneous abortions had a 70% greater risk (95% CI 1.27 to 2.27) (Table 3). Women who experienced induced abortion alone had an 18% (95% CI 1.07 to 1.31) greater risk of premature mortality from cancer. Additionally, women who experienced spontaneous abortion alone or induced abortion alone had a 29% (95% CI 1.06 to 1.58) and 47% (95% CI 1.16 to 1.85) greater risk of premature mortality from other causes, and the risk increased with the increasing number of spontaneous abortions (*P*_trend_ = 0.008). There was no evidence that the association for cause-specific premature death varied by HDP, BMI at baseline, smoking status, or endometriosis (Fig. 2 and Table S2).

The magnitude of associations in the imputed dataset and the other datasets in the sensitivity analyses was somewhat attenuated or stronger but remained statistically significant for all-cause and cause-specific premature death (Tables S3 to S6).

## Discussion

This nationwide prospective cohort study provides novel insights into disparities in premature death risk related to abortion among ever gravid women in the UK. We found that spontaneous abortion alone was associated with an increased risk of all-cause premature death when compared to no abortion history. Induced abortion alone was associated with an increased risk of all-cause premature death, particularly among women with HDP. Notably, this association was predominantly driven by an elevated risk of death from cardiovascular disease even after accounting for lifestyle, physical activity, diet score, and clinical conditions, which depended strongly on the number of induced abortions experienced over the reproductive period.

Most of previous studies have focused on maternal premature mortality risks associated with spontaneous abortions [19,28]. In the Nurses’ Health Study II of 101,681 ever gravid female nurses aged 25 to 42 years, spontaneous abortion was associated with an increased risk of premature mortality, particularly deaths from cardiovascular disease [19]. Data from the Collaborative Perinatal Project from 1959 to 1966 showed that spontaneous abortion was associated with a higher absolute risk of mortality from all causes and cardiovascular diseases [28]. Our findings were consistent with these previous studies by comprehensively delineating the associations between spontaneous abortion alone and increased risk of deaths from all causes but not from cardiovascular disease and cancer. Potential mechanisms underlying the association include the role of genetic or epigenetic factors [19]. Additionally, spontaneous abortion has been linked to increased risks of myocardial infarction and stroke, likely further contributing to an increased risk of maternal mortality [19,29]. Besides, chronic inflammation is common among women with diabetes, endometriosis, and polycystic ovary syndrome and was associated with higher risks of spontaneous abortion [28] and maternal mortality.

Previous studies of survival outcomes among women of reproductive age have shown mixed results, with reports of materially increased [19,30,31], unchanged [19,32], or decreased risks of death related to induced abortions [33]. A Danish study involving 463,473 women during a decade following pregnancy demonstrated that those who experienced induced abortions after 12 weeks’ gestation showed the highest mortality rates, followed by those who experienced induced abortions during early pregnancy, then by those with spontaneous abortions, with the lowest rates observed in those who had given birth [30]. A Finnish register-based cohort study between 1987 and 1994 showed that women experiencing induced abortions had a higher mortality risk compared to those who gave live births, as well as those experiencing spontaneous abortions or ectopic pregnancies [31]. Results from the same cohort with a follow-up of 14 years also showed a significantly elevated mortality rate in women with a history of induced abortion in comparison to non-pregnant women [20]. Nevertheless, some studies reported contrary results. For instance, in the Japan Collaborative Cohort Study of 54,652 women aged 40 to 79 years between 1988 and 1990, either spontaneous or induced abortion was associated with a lower risk of death from cardiovascular disease [33]. In the present study, our results indicated that induced abortion alone was associated with an increased risk of all-cause premature death and death from cardiovascular disease and cancer. The discrepancy across studies might be caused by the differences in sociodemographic characteristics, laws and policies on abortion restrictions, and medical conditions [19,34].

Since detailed reasons for induced abortion were not collected in this study, we could only make some speculations about the mechanisms that may explain its association with premature mortality. According to estimates from the WHO, unsafe abortions account for 45% of all abortions between 2010 and 2014, which approximately led to 13% of all maternal deaths worldwide [35,36]. Complications arising from unsafe abortion, such as hemorrhage, infection, and injury to the genital tract and internal organs, contribute to its grave impact on maternal mortality [37,38]. Additionally, studies have shown that up to 30% of women reported experiencing clinical levels of anxiety or high levels of general distress 3 to 4 weeks after induced abortion [39]. This observation is particularly important as distress has emerged as a potential risk factor for female mortality and mortality related to cardiovascular disease [40]. Alternatively, women who underwent abortions were more likely to have medical conditions, such as type 1 diabetes, which could render them susceptible to the onset of cardiovascular disease, ultimately contributing to an increased risk of mortality [41]. Notably, various reasons for induced abortions might also, to some extent, explain its effect on premature mortality. Firstly, low socioeconomic status, often associated with induced abortion, might limit access to healthcare, potentially increasing the risk of adverse consequences and mortality [42]. Secondly, maternal or fetal complications may necessitate induced abortions, thereby increasing the risk of mortality [43]. Additionally, ethical or religious beliefs might influence abortion decisions, potentially raising the risks of depression and anxiety, ultimately leading to mortality [44].

Furthermore, we also assessed the potential impact of baseline BMI, prior smoking status, endometriosis, and HDP on the association between abortion and the risk of premature death. Although these factors have been acknowledged as leading causes for mortality [34,45–47], evidence regarding their interaction with abortion in influencing the risk of premature death remains scarce and warrants further exploration. A systematic analysis, including 417 datasets from 115 countries worldwide, reported that HDP accounted for 14.0% of maternal deaths [34]. In our study, the results from stratified analysis revealed that the adverse effect of induced abortion alone could be amplified by the presence of HDP, with a statistically significant association observed. This finding highlights the importance of early evaluation and clinical supervision to preventing and controlling high blood pressure in pregnant women.

Our study has several strengths. First, it is one of the largest nationwide prospective studies investigating the association of abortion with premature mortality, complementing earlier studies conducted in the other countries including the United States [19], Finland [20], and Denmark [30]. Second, in order to better understand disease and its consequences, the UKB was devised to collect a wide range of essential confounders (e.g., smoking, diet score, physical activity, endometriosis, and ectopic pregnancy) that could bias the results to evaluate the associations. Additionally, this study also allowed us to conduct investigations into the potential modification effects of sociodemographic factors, lifestyle, and medical conditions on the associations between abortion and premature death.

Our study also has some limitations. First, the nature of observational cohort study precludes establishing causality. Second, self-reported data might yield misclassification bias, which should be considered when interpreting the results. Third, despite adjusting for various potential confounders, residual confounding cannot be precluded. Fourth, information regarding the specific reasons for induced abortions (e.g., mental factors, genetic disease, and ethical or religious beliefs) was not collected in this study, which prohibited us from exploring their impacts on premature mortality. In addition, our findings derived from the UK population aged 39 years and older might restrict the generalizability of the results to younger age groups; however, by excluding women aged 39 years and younger, and those who died before age 39 years, the risk of premature death might be overestimated.

### Conclusion

This nationwide prospective cohort study indicated that both spontaneous abortion and induced abortion alone were associated with an increased risk of premature mortality, with induced abortion alone particularly linked to death from cardiovascular disease. Future studies are encouraged to explore detailed reasons for abortions to facilitate mechanism investigation and to better inform strategies on reducing abortion-related health risks.

#### Ethical Approval

This study was approved by the UK North West Multi-Centre Research Ethics Committee (11/NW/0382) and all participants provided electronically signed informed consent before participating in the study.

## Data Availability

Access to data from UK Biobank (https://www.ukbiobank.ac.uk/) is available upon application.
