# Supplementary material for: Association between Abortion and All-Cause and Cause-Specific Premature Mortality: A Prospective Cohort Study from the UK Biobank
Source: Health Data Sci. 2024 Jul 15;4:0147. doi: 10.34133/hds.0147 (PMC11246836; doi:10.34133/hds.0147)
Supplement: Supplementary 1 — Tables S1 to S6 [file hds.0147.f1.docx]

**Association between abortion and all-cause and cause-specific premature mortality: a prospective cohort study from the UK Biobank**

Shaohua Yin; Yingying Yang; Qin Wang; Wei Guo; Qian He; Lei Yuan; Keyi Si

**Table S1. Hazard ratios (HRs) and 95% confidence intervals (CIs) for all-cause and cause-specific premature mortality (before age 70 years) among women aged ≥50 years, according to number of abortions**

|  |  | Crude model | | Multivariable model^*^ | |
| --- | --- | --- | --- | --- | --- |
|  | Cases/ Person-years | HR (95% CI) | P_-trend_ | HR (95% CI) | P_-trend_ |
| **All deaths** |  |  |  |  |  |
| Spontaneous abortion alone |  |  | <0.001 |  | <0.001 |
| None | 2,786/1,645,168 | 1.00 (ref) |  | 1.00 (ref) |  |
| 1 | 602/345,425 | 1.04 (0.95–1.14) |  | 1.02 (0.93–1.11) |  |
| 2 | 192/82,293 | **1.39 (1.20–1.61)** |  | **1.28 (1.11–1.49)** |  |
| ≥3 | 107/42,370 | **1.51 (1.24–1.83)** |  | **1.32 (1.09–1.60)** |  |
| Induced abortion alone |  |  | <0.001 |  | <0.001 |
| None | 2,786/1,645,168 | 1.00 (ref) |  | 1.00 (ref) |  |
| 1 | 468/214,132 | **1.35 (1.22–1.49)** |  | **1.33 (1.21–1.47)** |  |
| 2 | 80/35,211 | **1.44 (1.15–1.79)** |  | **1.41 (1.13–1.76)** |  |
| ≥3 | 20/8,204 | **1.58 (1.02–2.45)** |  | **1.61 (1.04–2.51)** |  |
| Spontaneous and induced abortion |  |  | 0.027 |  | 0.684 |
| None | 2,786/1,645,168 | 1.00 (ref) |  | 1.00 (ref) |  |
| 2 | 97/56,213 | 1.07 (0.88–1.31) |  | 0.92 (0.75–1.13) |  |
| ≥3 | 86/40,621 | **1.33 (1.08–1.65)** |  | 1.03 (0.83–1.28) |  |
| **Deaths from cardiovascular disease** |  |  |  |  |  |
| Spontaneous abortion alone |  |  | <0.001 |  | <0.001 |
| None | 762/1,631,730 | 1.00 (ref) |  | 1.00 (ref) |  |
| 1 | 149/342,356 | 0.94 (0.79–1.12) |  | 0.92 (0.77–1.10) |  |
| 2 | 53/81,403 | **1.41 (1.06–1.86)** |  | 1.26 (0.95–1.66) |  |
| ≥3 | 43/41,951 | **2.22 (1.64–3.02)** |  | **1.83 (1.34–2.49)** |  |
| Induced abortion alone |  |  | <0.001 |  | 0.047 |
| None | 762/1,631,730 | 1.00 (ref) |  | 1.00 (ref) |  |
| 1 | 141/211,830 | **1.49 (1.25–1.79)** |  | **1.27 (1.06–1.53)** |  |
| 2 | 20/34,811 | 1.32 (0.85–2.06) |  | 1.01 (0.64–1.58) |  |
| ≥3 | 7/8,105 | 2.05 (0.97–4.30) |  | **1.60 (1.00–3.39)** |  |
| Spontaneous and induced abortion |  |  | 0.236 |  | 0.377 |
| None | 762/1,631,730 | 1.00 (ref) |  | 1.00 (ref) |  |
| 2 | 21/55,685 | 0.85 (0.55–1.32) |  | 0.74 (0.48–1.14) |  |
| ≥3 | 24/40,190 | 1.37 (0.91–2.06) |  | 1.04 (0.69–1.57) |  |
| **Deaths from cancer** |  |  |  |  |  |
| Spontaneous abortion alone |  |  | 0.127 |  | 0.060 |
| None | 1,749/1,637,764 | 1.00 (ref) |  | 1.00 (ref) |  |
| 1 | 378/343,863 | 1.04 (0.93–1.16) |  | 1.01 (0.91–1.14) |  |
| 2 | 120/81,769 | **1.39 (1.15–1.67)** |  | **1.29 (1.07–1.56)** |  |
| ≥3 | 50/41,914 | 1.12 (0.85–1.49) |  | 1.01 (0.76–1.34) |  |
| Induced abortion alone |  |  | <0.001 |  | 0.001 |
| None | 1,749/1,637,764 | 1.00 (ref) |  | 1.00 (ref) |  |
| 1 | 270/212,594 | **1.24 (1.09–1.40)** |  | **1.20 (1.05–1.36)** |  |
| 2 | 54/35,006 | **1.54 (1.17–2.02)** |  | **1.49 (1.14–1.96)** |  |
| ≥3 | 11/8,135 | 1.37 (0.76–2.49) |  | 1.42 (0.78–2.57) |  |
| Spontaneous and induced abortion |  |  | 0.102 |  | 0.967 |
| None | 1,749/1,637,764 | 1.00 (ref) |  | 1.00 (ref) |  |
| 2 | 65/55,950 | 1.14 (0.89–1.46) |  | 0.96 (0.48–1.14) |  |
| ≥3 | 53/40,362 | 1.30 (0.99–1.71) |  | 0.99 (0.69–1.57) |  |
| **Deaths from all other causes** |  |  |  |  |  |
| Spontaneous abortion alone |  |  | 0.010 |  | 0.034 |
| None | 273/1,628,309 | 1.00 (ref) |  | 1.00 (ref) |  |
| 1 | 75/341,824 | **1.33 (1.03–1.71)** |  | **1.32 (1.02–1.70)** |  |
| 2 | 19/81,162 | 1.41 (0.88–2.24) |  | 1.30 (0.82–2.07) |  |
| ≥3 | 14/41,714 | **2.02 (1.18–3.45)** |  | **1.78 (1.04–3.06)** |  |
| Induced abortion alone |  |  | 0.732 |  | 0.117 |
| None | 273/1,628,309 | 1.00 (ref) |  | 1.00 (ref) |  |
| 1 | 57/211,143 | **1.68 (1.26–2.23)** |  | **1.41 (1.05–1.88)** |  |
| 2 | 6/34,680 | 1.10 (0.49–2.47) |  | 0.79 (0.35–1.80) |  |
| ≥3 | 2/8,079 | 1.61 (0.40–6.48) |  | 1.16 (0.29–4.71) |  |
| Spontaneous and induced abortion |  |  | 0.463 |  | 0.915 |
| None | 273/1,628,309 | 1.00 (ref) |  | 1.00 (ref) |  |
| 2 | 11/55,595 | 1.24 (0.68–2.27) |  | 1.08 (0.59–1.99) |  |
| ≥3 | 9/40,051 | 1.43 (0.73–2.77) |  | 1.12 (0.58–2.19) |  |

HR=hazard ratio. 95% CI=95% confidence interval. Bold indicates the p-values less than 0.05 were considered as statistically significant.

* Multivariable models were adjusted for age at baseline, BMI at baseline, ethnicity, education, average household income, smoking status, drinking status, diabetes at baseline, cancer at baseline, use of oral contraceptives, use of hormone treatment, menopausal status, gestational diabetes, hypertensive disorders of pregnancy, hemorrhage during early pregnancy, endometriosis, ectopic pregnancy, parental history of myocardial infarction or stroke, physical activity, and diet score.

**Table S2. Adjusted hazard ratios (HRs) and 95% confidence intervals (CI) for premature mortality according to method of abortion, stratified by BMI at baseline, prior smoking status, and** **endometriosis**

|  | Adjusted HRs (95% CI)^*^ | | | |
| --- | --- | --- | --- | --- |
| **Stratified factors** | No abortion history | Spontaneous abortion alone | Induced abortion alone | Spontaneous and induced abortion |
| **All deaths** |  |  |  |  |
| **BMI at baseline (kg/m^2^)** |  |  |  |  |
| <18.5 | 1.00 (ref) | 0.68 (0.33–1.42) | 1.27 (0.62–2.58) | 1.39 (0.46–4.13) |
| 18.5–24.9 | 1.00 (ref) | **1.14 (1.01–1.28)** | 1.11 (0.96–1.27) | 1.15 (0.93–1.41) |
| 25–29.9 | 1.00 (ref) | **1.13 (1.01–1.27)** | **1.18 (1.03–1.36)** | 1.04 (0.83–1.31) |
| ≥30 | 1.00 (ref) | 1.07 (0.95–1.22) | **1.22 (1.05–1.41)** | 1.08 (0.86–1.36) |
| Missing | 1.00 (ref) | 0.94 (0.53–1.65) | 1.11 (0.55–2.25) | 1.87 (0.74–4.72) |
| P for multiplicative interaction | 0.957 |  |  |  |
| **Prior smoking status** |  |  |  |  |
| No | 1.00 (ref) | 1.08 (0.96–1.21) | 1.11 (0.95–1.29) | 1.08 (0.85–1.36) |
| Yes | 1.00 (ref) | **1.12 (1.03–1.22)** | **1.20 (1.09–1.32)** | 1.12 (0.97–1.30) |
| Missing | 1.00 (ref) | 1.08 (0.43–2.70) | 0.35 (0.05–2.68) | 0.70 (0.08–5.82) |
| P for multiplicative interaction | 0.859 |  |  |  |
| **Endometriosis** |  |  |  |  |
| Without endometriosis | 1.00 (ref) | **1.09 (1.02–1.17)** | **1.16 (1.06–1.25)** | 1.11 (0.98–1.26) |
| With endometriosis | 1.00 (ref) | **1.60 (1.11–2.30)** | 1.54 (0.97–2.42) | 1.01 (0.48–2.11) |
| P for multiplicative interaction | 0.247 |  |  |  |
| **Deaths from cardiovascular disease** | |  |  |  |
| **BMI at baseline (kg/m^2^)** |  |  |  |  |
| <18.5 | 1.00 (ref) | 1.39 (0.22–8.68) | 2.40 (0.4–14.22) | **8.49 (1.15–62.68)** |
| 18.5–24.9 | 1.00 (ref) | 1.07 (0.82–1.38) | 1.05 (0.77–1.42) | 1.06 (0.67–1.68) |
| 25–29.9 | 1.00 (ref) | 1.09 (0.86–1.37) | 1.26 (0.96–1.65) | 0.62 (0.34–1.10) |
| ≥30 | 1.00 (ref) | 1.04 (0.83–1.29) | **1.24 (1.01–1.60)** | 1.19 (0.82–1.72) |
| Missing | 1.00 (ref) | 0.25 (0.06–1.04) | 0.57 (0.13–2.46) | 1.25 (0.23–6.86) |
| P for multiplicative interaction | 0.309 |  |  |  |
| **Prior smoking status** |  |  |  |  |
| No | 1.00 (ref) | 0.98 (0.78–1.24) | 1.11 (0.83–1.50) | 0.94 (0.57–1.53) |
| Yes | 1.00 (ref) | 1.07 (0.91–1.26) | **1.22 (1.01–1.46)** | 1.05 (0.78–1.40) |
| Missing | 1.00 (ref) | 1.59 (0.39–6.44) | 0.62 (0.05–7.54) | NA |
| P for multiplicative interaction | 0.979 |  |  |  |
| **Endometriosis** |  |  |  |  |
| Without endometriosis | 1.00 (ref) | 1.01 (0.88–1.16) | **1.19 (1.01–1.39)** | 1.04 (0.81–1.33) |
| With endometriosis | 1.00 (ref) | **1.99 (1.06–3.71)** | 1.11 (0.41–3.01) | 0.52 (0.07–3.9) |
| P for multiplicative interaction | 0.121 |  |  |  |
| **Deaths from cancer** | |  |  |  |
| **BMI at baseline (kg/m^2^)** |  |  |  |  |
| <18.5 | 1.00 (ref) | 0.43 (0.15–1.22) | 1.06 (0.42–2.62) | 0.69 (0.14–3.29) |
| 18.5–24.9 | 1.00 (ref) | 1.13 (0.98–1.31) | 1.11 (0.94–1.31) | 1.14 (0.88–1.48) |
| 25–29.9 | 1.00 (ref) | 1.13 (0.97–1.30) | 1.12 (0.94–1.33) | 1.16 (0.89–1.51) |
| ≥30 | 1.00 (ref) | 1.08 (0.91–1.28) | 1.16 (0.94–1.42) | 1.01 (0.73–1.38) |
| Missing | 1.00 (ref) | 0.93 (0.32–2.73) | 1.02 (0.25–4.14) | 2.55 (0.51–12.8) |
| P for multiplicative interaction | 0.992 |  |  |  |
| **Prior smoking status** |  |  |  |  |
| No | 1.00 (ref) | 1.14 (0.98–1.31) | 1.07 (0.88–1.30) | 1.06 (0.78–1.44) |
| Yes | 1.00 (ref) | 1.10 (0.98–1.22) | **1.16 (1.02–1.30)** | 1.14 (0.94–1.37) |
| Missing | 1.00 (ref) | 0.37 (0.04–3.70) | NA | 4.70 (0.27–81.66) |
| P for multiplicative interaction | 0.893 |  |  |  |
| **Endometriosis** |  |  |  |  |
| Without endometriosis | 1.00 (ref) | **1.10 (1.01–1.20)** | **1.11 (1.00–1.23)** | 1.12 (0.95–1.31) |
| With endometriosis | 1.00 (ref) | 1.31 (0.82–2.11) | 1.56 (0.90–2.70) | 1.05 (0.44–2.48) |
| P for multiplicative interaction | 0.737 |  |  |  |
| **Deaths from all other causes** | |  |  |  |
| **BMI at baseline (kg/m^2^)** |  |  |  |  |
| <18.5 | 1.00 (ref) | 1.23 (0.29–5.20) | 1.40 (0.24–8.05) | NA |
| 18.5–24.9 | 1.00 (ref) | 1.30 (0.92–1.83) | 1.25 (0.84–1.86) | 1.34 (0.75–2.39) |
| 25–29.9 | 1.00 (ref) | 1.34 (0.91–1.96) | 1.46 (0.93–2.27) | 1.43 (0.71–2.85) |
| ≥30 | 1.00 (ref) | 1.15 (0.79–1.67) | 1.47 (0.96–2.23) | 1.10 (0.55–2.20) |
| Missing | 1.00 (ref) | 1.65 (0.63–4.33) | 1.58 (0.51–4.93) | 3.00 (0.57–15.76) |
| P for multiplicative interaction | 0.999 |  |  |  |
| **Prior smoking status** |  |  |  |  |
| No | 1.00 (ref) | 1.04 (0.74–1.46) | 1.29 (0.85–1.97) | 1.50 (0.82–2.72) |
| Yes | 1.00 (ref) | **1.43 (1.11–1.85)** | **1.45 (1.09–1.93)** | 1.24 (0.79–1.96) |
| Missing | 1.00 (ref) | 1.35 (0.01–142.43) | NA | NA |
| P for multiplicative interaction | 0.772 |  |  |  |
| **Endometriosis** |  |  |  |  |
| Without endometriosis | 1.00 (ref) | **1.26 (1.03–1.54)** | **1.34 (1.06–1.70)** | 1.28 (0.89–1.84) |
| With endometriosis | 1.00 (ref) | 4.08 (0.87–19.11) | **6.17 (1.17–32.48)** | 2.71 (0.25–28.81) |
| P for multiplicative interaction | 0.449 |  |  |  |

HR=hazard ratio. 95% CI=95% confidence interval. Bold indicates the p-values less than 0.05 were considered as statistically significant.

* Multivariable models were adjusted for age at baseline, BMI at baseline, ethnicity, education, average total household income, smoking status, drinking status, diabetes at baseline, cancer at baseline, use of oral contraceptives, use of hormone treatment, menopausal status, gestational diabetes, hypertensive disorders of pregnancy, hemorrhage during early pregnancy, endometriosis, ectopic pregnancy, parental history of myocardial infarction or stroke, physical activity, and diet score.

**Table S3. Hazard ratios (HRs) and 95% confidence intervals (CI) for all-cause and cause-specific premature mortality, according to method of abortion, using multiple imputation approach**

|  |  | | Crude model | | Multivariable model^*^ | |
| --- | --- | --- | --- | --- | --- | --- |
|  | Cases/ Person-years | | HR (95% CI) | AR% | HR (95% CI) | AR% |
| **All deaths** | |  |  |  |  |  |
| No abortion history | | 3,231/2,032,953 | 1.00 (ref) |  | 1.00 (ref) |  |
| Spontaneous abortion alone | | 1,094/612,687 | **1.12 (1.05–1.20)** | 11.03 | **1.14 (1.11–1.18)** | 12.28 |
| Induced abortion alone | | 757/381710 | **1.25 (1.15–1.35)** | 19.81 | **1.35 (1.30–1.40)** | 25.87 |
| Spontaneous and induced abortion | | 271/147,107 | **1.16 (1.02–1.31)** | 13.64 | **1.25 (1.19–1.33)** | 20.26 |
| **Deaths from cardiovascular disease** | |  |  |  |  |  |
| No abortion history | | 885/2,016,721 | 1.00 (ref) |  | 1.00 (ref) |  |
| Spontaneous abortion alone | | 286/606,938 | 1.07 (0.94–1.23) | 6.72 | **1.08 (1.02–1.15)** | 7.49 |
| Induced abortion alone | | 205/377,541 | **1.24 (1.06–1.44)** | 19.03 | **1.42 (1.32–1.52)** | 29.48 |
| Spontaneous and induced abortion | | 68/145,574 | 1.06 (0.83–1.36) | 5.93 | 1.23 (0.98–1.37) | 18.63 |
| **Deaths from cancer** | |  |  |  |  |  |
| No abortion history | | 2,010/2,023,840 | 1.00 (ref) |  | 1.00 (ref) |  |
| Spontaneous abortion alone | | 676/609,532 | **1.12 (1.02–1.22)** | 10.71 | **1.14 (1.09–1.18)** | 12.13 |
| Induced abortion alone | | 458/379,328 | **1.21 (1.09–1.34)** | 17.36 | **1.29 (1.23–1.35)** | 22.60 |
| Spontaneous and induced abortion | | 170/146,233 | 1.17 (1.00–1.36) | 14.53 | **1.26 (1.17–1.35)** | 20.32 |
| **Deaths from all other causes** | |  |  |  |  |  |
| No abortion history | | 336/2,012,686 | 1.00 (ref) |  | 1.00 (ref) |  |
| Spontaneous abortion alone | | 132/605,728 | **1.30 (1.07–1.59)** | 23.31 | **1.33 (1.21–1.45)** | 24.64 |
| Induced abortion alone | | 94/376,586 | **1.49 (1.19–1.87)** | 32.89 | **1.62 (1.46–1.80)** | 38.27 |
| Spontaneous and induced abortion | | 33/145,223 | 1.36 (0.95–1.94) | 26.31 | **1.48 (1.26–1.74)** | 32.61 |

AR%=attributable risk proportion. HR=hazard ratio. 95% CI=95% confidence interval. Bold indicates the p-values less than 0.05 were considered as statistically significant.

* Multivariable models were adjusted for age at baseline, BMI at baseline, ethnicity, education, average total household income, smoking status, drinking status, diabetes at baseline, cancer at baseline, use of oral contraceptives, use of hormone treatment, menopausal status, gestational diabetes, hypertensive disorders of pregnancy, hemorrhage during early pregnancy, endometriosis, ectopic pregnancy, parental history of myocardial infarction or stroke, physical activity, and diet score. Multiple imputation was performed for all independent variables included in the analysis which had missing data (BMI at baseline, ethnicity, education, average total household income, smoking status, drinking status, diabetes at baseline, cancer at baseline, use of oral contraceptives, use of hormone treatment, menopausal status, gestational diabetes, physical activity, and diet score).

**Table S4. Hazard ratios (HRs) and 95% confidence intervals (CI) for all-cause and cause-specific premature mortality among women without cancer at baseline, according to method of abortion**

|  |  | | Crude model | | Multivariable model^*^ | |
| --- | --- | --- | --- | --- | --- | --- |
|  | Cases/ Person-years | | HR (95% CI) | AR% | HR (95% CI) | AR% |
| **All deaths** | |  |  |  |  |  |
| No abortion history | | 2,451/1,848,538 | 1.00 (ref) |  | 1.00 (ref) |  |
| Spontaneous abortion alone | | 834/555,530 | **1.19 (1.10–1.29)** | 16.25 | **1.13 (1.04–1.22)** | 11.19 |
| Induced abortion alone | | 583/348,384 | **1.52 (1.39–1.66)** | 34.17 | **1.19 (1.09–1.31)** | 15.97 |
| Spontaneous and induced abortion | | 207/134,217 | **1.44 (1.25–1.65)** | 30.36 | 1.12 (0.97–1.29) | 10.71 |
| **Deaths from cardiovascular disease** | |  |  |  |  |  |
| No abortion history | | 743/1,835,943 | 1.00 (ref) |  | 1.00 (ref) |  |
| Spontaneous abortion alone | | 238/550,975 | 1.13 (0.97–1.30) | 11.19 | 1.11 (0.96–1.28) | 9.67 |
| Induced abortion alone | | 177/345,107 | **1.54 (1.30–1.81)** | 34.85 | **1.57 (1.33–1.85)** | 36.14 |
| Spontaneous and induced abortion | | 56/132,977 | 1.30 (0.99–1.70) | 22.78 | 1.30 (0.99–1.71) | 23.14 |
| **Deaths from cancer** | |  |  |  |  |  |
| No abortion history | | 1,405/1,840,610 | 1.00 (ref) |  | 1.00 (ref) |  |
| Spontaneous abortion alone | | 479/552,773 | **1.20 (1.08–1.33)** | 16.32 | **1.16 (1.05–1.29)** | 14.09 |
| Induced abortion alone | | 318/346,254 | **1.44 (1.28–1.63)** | 30.60 | **1.26 (1.12–1.43)** | 20.89 |
| Spontaneous and induced abortion | | 122/133,447 | **1.47 (1.22–1.77)** | 32.07 | **1.28 (1.06–1.54)** | 21.88 |
| **Deaths from all other causes** | |  |  |  |  |  |
| No abortion history | | 303/1,832,587 | 1.00 (ref) |  | 1.00 (ref) |  |
| Spontaneous abortion alone | | 117/549,960 | **1.35 (1.09–1.67)** | 25.98 | **1.30 (1.05–1.62)** | 23.25 |
| Induced abortion alone | | 88/344,323 | **1.84 (1.45–2.33)** | 45.53 | **1.65 (1.30–2.10)** | 39.43 |
| Spontaneous and induced abortion | | 29/132,681 | **1.61 (1.10–2.36)** | 37.85 | 1.47 (1.00–2.15) | 31.74 |

AR%=attributable risk proportion. HR=hazard ratio. 95% CI=95% confidence interval. Bold indicates the p-values less than 0.05 were considered as statistically significant.

* Multivariable models were adjusted for age at baseline, BMI at baseline, ethnicity, education, average total household income, smoking status, drinking status, diabetes at baseline, use of oral contraceptives, use of hormone treatment, menopausal status, gestational diabetes, hypertensive disorders of pregnancy, hemorrhage during early pregnancy, endometriosis, ectopic pregnancy, parental history of myocardial infarction or stroke, physical activity, and diet score.

**Table S5. Hazard ratios (HRs) and 95% confidence intervals (CI) for all-cause and cause-specific premature mortality among women with at least one live birth, according to method of abortion**

|  |  | | | Crude model | | | Multivariable model^*^ | | | |
| --- | --- | --- | --- | --- | --- | --- | --- | --- | --- | --- |
|  | Cases/ Person-years | | | HR (95% CI) | | AR% | HR (95% CI) | | AR% | |
| **All deaths** | |  |  | |  | | |  | |  |
| No abortion history | | 2,570/1,700,539 | 1.00 (ref) | |  | | | 1.00 (ref) | |  |
| Spontaneous abortion alone | | 828/494,706 | **1.16 (1.07–1.25)** | | 13.79 | | | **1.10 (1.01–1.19)** | | 9.09 |
| Induced abortion alone | | 421/233,463 | **1.37 (1.24–1.52)** | | 27.01 | | | **1.11 (1.00–1.24)** | | 9.91 |
| Spontaneous and induced abortion | | 180/107,379 | **1.33 (1.14–1.55)** | | 24.81 | | | 1.06 (0.91–1.23) | | 5.66 |
| **Deaths from cardiovascular disease** | |  |  | |  | | |  | |  |
| No abortion history | | 66/1,687,450 | 1.00 (ref) | |  | | | 1.00 (ref) | |  |
| Spontaneous abortion alone | | 20/490,274 | 1.11 (0.94–1.29) | | 9.91 | | | 1.03 (0.88–1.21) | | 2.91 |
| Induced abortion alone | | 117/231,174 | **1.49 (1.23–1.82)** | | 32.89 | | | **1.23 (1.01–1.50)** | | 18.70 |
| Spontaneous and induced abortion | | 43/106,356 | 1.25 (0.92–1.70) | | 20.00 | | | 0.99 (0.72–1.35) | | –1.01 |
| **Deaths from cancer** | |  |  | |  | | |  | |  |
| No abortion history | | 1,646/1,693,839 | 1.00 (ref) | |  | | | 1.00 (ref) | |  |
| Spontaneous abortion alone | | 52/492,418 | **1.14 (1.03–1.26)** | | 12.28 | | | 1.10 (0.99–1.21) | | 9.09 |
| Induced abortion alone | | 25/232,158 | **1.27 (1.12–1.46)** | | 21.26 | | | 1.04 (0.91–1.19) | | 3.85 |
| Spontaneous and induced abortion | | 118/106,860 | **1.36 (1.13–1.64)** | | 26.47 | | | 1.09 (0.90–1.31) | | 8.26 |
| **Deaths from all other causes** | |  |  | |  | | |  | |  |
| No abortion history | | 262/1,684,652 | 1.00 (ref) | |  | | | 1.00 (ref) | |  |
| Spontaneous abortion alone | | 102/489,480 | **1.40 (1.11–1.76)** | | 28.57 | | | 1.30 (1.04–1.64) | | 23.08 |
| Induced abortion alone | | 53/230,648 | **1.69 (1.26–2.27)** | | 40.83 | | | 1.37 (1.01–1.84) | | 27.01 |
| Spontaneous and induced abortion | | 19/106,132 | 1.37 (0.86–2.18) | | 27.01 | | | 1.09 (0.68–1.73) | | 8.26 |

AR%=attributable risk proportion. HR=hazard ratio. 95% CI=95% confidence interval. Bold indicates the p-values less than 0.05 were considered as statistically significant.

* Multivariable models were adjusted for age at baseline, BMI at baseline, ethnicity, education, average total household income, smoking status, drinking status, diabetes at baseline, cancer at baseline, use of oral contraceptives, use of hormone treatment, menopausal status, gestational diabetes, hypertensive disorders of pregnancy, hemorrhage during early pregnancy, endometriosis, ectopic pregnancy, parental history of myocardial infarction or stroke, physical activity, and diet score.

**Table S6. Hazard ratios (HRs) and 95% confidence intervals (CI) for all-cause and cause-specific premature mortality among women aged ≥50 years, according to method of abortion**

|  |  | | Crude model | | Multivariable model^*^ | |
| --- | --- | --- | --- | --- | --- | --- |
|  | Cases/ Person-years | | HR (95% CI) | AR% | HR (95% CI) | AR% |
| **All deaths** | |  |  |  |  |  |
| No abortion history | | 2,786/1,645,168 | 1.00 (ref) |  | 1.00 (ref) |  |
| Spontaneous abortion alone | | 901/47,008 | **1.15 (1.06–1.23)** | 13.04 | **1.09 (1.01–1.18)** | 8.26 |
| Induced abortion alone | | 568/257,546 | **1.37 (1.25–1.50)** | 27.01 | **1.11 (1.01–1.21)** | 9.91 |
| Spontaneous and induced abortion | | 183/96,834 | **1.18 (1.02–1.37)** | 15.25 | 0.97 (0.83–1.13) | -3.09 |
| **Deaths from** **cardiovascular disease** | |  |  |  |  |  |
| No abortion history | | 762/1,631,730 | 1.00 (ref) |  | 1.00 (ref) |  |
| Spontaneous abortion alone | | 245/465,710 | 1.14 (0.99–1.32) | 12.28 | 1.07 (0.93–1.24) | 8.41 |
| Induced abortion alone | | 168/254,746 | **1.49 (1.26–1.76)** | 32.88 | **1.46 (1.04–1.62)** | 31.51 |
| Spontaneous and induced abortion | | 45/95,875 | 1.07 (0.79–1.44) | 6.54 | 0.87 (0.64–1.18) | -14.94 |
| **Deaths from cancer** | |  |  |  |  |  |
| No abortion history | | 1,749/1,637,764 | 1.00 (ref) |  | 1.00 (ref) |  |
| Spontaneous abortion alone | | 548/467,546 | **1.11 (1.01–1.22)** | 9.91 | 1.06 (0.97–1.17) | 5.66 |
| Induced abortion alone | | 335/255,736 | **1.28 (1.14–1.44)** | 21.88 | **1.03 (1.01–1.14)** | 2.91 |
| Spontaneous and induced abortion | | 118/96,312 | 1.21 (1.00–1.45) | 17.36 | 0.99 (0.82–1.19) | -1.01 |
| **Deaths from all other causes** | |  |  |  |  |  |
| No abortion history | | 273/1,628,309 | 1.00 (ref) |  | 1.00 (ref) |  |
| Spontaneous abortion alone | | 108/464,699 | **1.40 (1.12–1.75)** | 28.57 | **1.36 (1.08–1.69)** | 26.47 |
| Induced abortion alone | | 65/253,902 | **1.60 (1.22–2.10)** | 37.50 | **1.32 (1.01–1.74)** | 24.24 |
| Spontaneous and induced abortion | | 20/95,646 | 1.32 (0.84–2.07) | 24.24 | 1.13 (0.71–1.78) | 11.50 |

AR%=attributable risk proportion. HR=hazard ratio. Bold indicates the p-values less than 0.05 were considered as statistically significant.

* Multivariable models were adjusted for age at baseline, BMI at baseline, ethnicity, education, average household income, smoking status, drinking status, diabetes at baseline, cancer at baseline, use of oral contraceptives, use of hormone treatment, menopausal status, gestational diabetes, hypertensive disorders of pregnancy, hemorrhage during early pregnancy, endometriosis, ectopic pregnancy, parental history of myocardial infarction or stroke, physical activity, and diet score.
